# Characterization of gross genome rearrangements in *Deinococcus radiodurans recA* mutants

**DOI:** 10.1038/s41598-021-89173-9

**Published:** 2021-05-25

**Authors:** Jelena Repar, Davor Zahradka, Ivan Sović, Ksenija Zahradka

**Affiliations:** 1 Laboratory for Molecular Microbiology, Division of Molecular Biology, Ruđer Bošković Institute, Bijenička cesta 54, 10000 Zagreb, Croatia; 2 Digital BioLogic d.o.o, Ivanić-Grad, Croatia

**Keywords:** Genomic instability, Genomics, Microbiology, Microbial genetics, Bacterial genetics, Genetics, Next-generation sequencing, DNA damage and repair, Double-strand DNA breaks

## Abstract

Genome stability in radioresistant bacterium *Deinococcus radiodurans* depends on RecA, the main bacterial recombinase. Without RecA, gross genome rearrangements occur during repair of DNA double-strand breaks. Long repeated (insertion) sequences have been identified as hot spots for ectopic recombination leading to genome rearrangements, and single-strand annealing (SSA) postulated to be the most likely mechanism involved in this process. Here, we have sequenced five isolates of *D. radiodurans recA* mutant carrying gross genome rearrangements to precisely characterize the rearrangements and to elucidate the underlying repair mechanism. The detected rearrangements consisted of large deletions in chromosome II in all the sequenced *recA* isolates. The mechanism behind these deletions clearly differs from the classical SSA; it utilized short (4–11 bp) repeats as opposed to insertion sequences or other long repeats. Moreover, it worked over larger linear DNA distances from those previously tested. Our data are most compatible with alternative end-joining, a recombination mechanism that operates in eukaryotes, but is also found in *Escherichia coli*. Additionally, despite the *recA* isolates being preselected for different rearrangement patterns, all identified deletions were found to overlap in a 35 kb genomic region. We weigh the evidence for mechanistic vs. adaptive reasons for this phenomenon.

## Introduction

*Deinococcus radiodurans* is a bacterium capable of surviving extreme quantities of DNA double-strand breaks (DSBs) caused by agents such as γ-radiation and desiccation^1–3^. Whereas one unrepaired DSB in the genome is expected to affect cell viability, *D. radiodurans* can reassemble its genome with great accuracy after hundreds of simultaneous DSBs^4,5^. The specific repair mechanism of *D. radiodurans*, the extended synthesis-dependent strand annealing (ESDSA), depends on the RecA protein, the main bacterial recombinase^6^. ESDSA involves extensive DNA end resection resulting in the long stretches of single-stranded DNA, presumably enabling the subsequent homologous pairing to bridge any long repetitive sequences and thus avoid genome rearrangements^6^. Indeed, the *D. radiodurans* genome does not lack repetitive sequences when compared to genetically close, non-radiation-resistant species^7^, being rich in both insertions sequences and also in shorter repeats^8,9^. Investigating the fidelity of *D. radiodurans* DSB repair, we have previously identified conditions under which the accuracy of *D. radiodurans* DNA repair is impaired resulting in gross genome rearrangements; this genome instability was present in *recA* mutants, and also in wild-type (wt) cells irradiated with extremely high doses of γ-radiation^10^.

*D. radiodurans recA* mutants are very sensitive to γ-radiation and prone to genomic structural change both after spontaneous and radiation-induced DSBs^10^. Spontaneous DSBs occasionally occur during DNA replication. Collapsed or broken replication forks may provide free double-strand ends that must be properly processed by DNA repair enzymes to preserve chromosomal integrity and resume DNA replication^11^. In *D. radiodurans* cells that are devoid of the RecA recombinase, such DNA ends are a likely source of chromosome instability. The absence of RecA protein strongly affects growth and viability of *D. radiodurans* cells, presumably because of their inability to efficiently repair spontaneous DSBs^3,10^.

Despite lacking an important DSB repair protein, the *D. radiodurans recA* mutants are able to recover longer fragments of DNA from shorter ones at high, genome-shattering, doses of γ-radiation^6,12,13^. This RecA-independent DSB repair has been proposed to occur through single-strand annealing (SSA)^6,12,13^, a mechanism that is expected to be less accurate, i.e. more easily misled by repetitive sequences, than the homologous recombination (HR) mechanisms that depend on the longer stretches of DNA^14,15^. Indeed, the experiments with *recA* mutants carrying various genetically engineered reporter systems with repetitive sequences have provided strong evidence for SSA as a major RecA-independent mechanism of DSB repair in *D. radiodurans*^12,16,17^. Formally, the RecA-independent DSB repair could also be carried out through the mechanism of non-homologous end-joining (NHEJ) which requires little or no homology between two ends exposed by DSB, and therefore, it is not specifically limited to any region of the genome^18,19^. NHEJ is one of the major pathways that repairs DSBs in eukaryotes but has also been found to operate in some prokaryotes, including *Mycobacteria* and *Bacillus subtilis*^20^. However, genetic studies conducted so far have failed to detect NHEJ-like recombination events in *D. radiodurans* suggesting that random end-to-end joining during DSB repair is extremely rare, if not absent, in this bacterium^6,10,12,21^.

Protection of proteins from oxidative stress in *D. radiodurans*, including, presumably, DNA repair proteins, plays an important role in surviving γ-radiation^22–25^. This protection seems to be mediated by Mn^2+^ ions in complex with small metabolites^22,26^ and starts to fail at very high doses of γ-radiation^24^. Very high doses of γ-radiation (25 kGy) cause genome instability in the *D. radiodurans* wt^10^. Interestingly, at the same level of protein damage (at 3.2 kGy), albeit at the much lower level of DSBs, the genome of the radiation-sensitive *Escherichia coli* may also experience some structural change^27^. Despite the strong oxidative stress protection, it is possible that proteins of *D. radiodurans* are adversely affected at the dose as high as 25 kGy, or that the ESDSA proteins (including RecA) are saturated by the numerous DSBs^10^, as the number of DSBs grows proportionally with the radiation dose. Both scenarios would result in a greater deployment of RecA-independent repair which is more prone to genome rearrangements^10^.

DSBs are cytotoxic lesions that threaten genomic stability and integrity. In eukaryotes, gross genome rearrangements triggered by DSBs can lead to severe diseases with propensity to cancer, premature aging, immune dysfunction, and neurological degeneration^28–30^. Genome rearrangements, except for inversions symmetrical around the *ori*-*ter* axis^31^, are expected to be deleterious in prokaryotes due to the disruption of genome organization^32,33^. Genome rearrangements can also have a direct effect on the gene dose through copy number change of a chromosome region and can disrupt genes or their regulation by introducing new breakpoints. Additionally, as all genome changes, genome rearrangements are a potential source of variability in population. Here, we report sequenced and assembled genomes of *D. radiodurans recA* isolates carrying gross genome rearrangements. We characterize the types of genome changes that take place in the *recA* mutants, discuss their phenotype implications and provide new information on the RecA-independent mechanism of DSB repair in *D. radiodurans*.

## Materials and methods

### Bacterial strains and growth conditions

**Figure 1:**
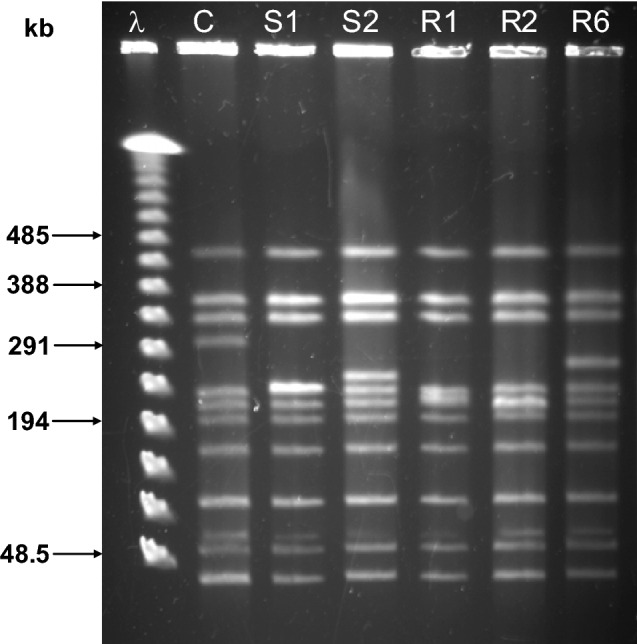
DNA of five *recA* isolates digested with NotI restriction enzyme and visualised by pulsed-field gel electrophoresis. S1 and S2 isolates have been obtained during normal lab propagation, and R1, R2 and R6 have been obtained after γ-radiation (see “Materials and methods”). The *recA* strain N1 with wt-like NotI pattern was used as control (C) and λ ladder as a size marker. Different NotI patterns were used as a prerequisite for the choice of isolates to be sequenced.

*D. radiodurans* strains used were the R1 wild type (wt, ATCC 13939), and its *recA* isolates derived from the GY10929 Δ *(cinA ligT recA)::tet*^34^. Spontaneous genome rearrangements were detected through the lab propagation of the GY10929 strain (isolates S1 and S2) and after 5 kGy of γ-radiation (isolates R1 and R2). In addition, the GY10929 strain was reconstructed by the transfer of Δ *(cinA ligT recA)::tet* cassette into the *D. radiodurans* wild type and named N1. A rearranged isolate R6 was obtained with the same protocol as R1 and R2, from the N1 strain. Repeated occurrence of discrete DNA rearrangements in *D. radiodurans recA* isolates was observed previously^10^. The isolates for sequencing (named S1, S2, R1, R2 and R6) were chosen based on the diversity of their rearrangement types—restriction enzyme NotI was used for the visualisation of rearrangements by pulsed-field gel electrophoresis (PFGE) (Fig. 1). In addition, the selected *recA* isolates showed improved γ-survival compared to the control *recA* strain (Figure S1). PFGE analysis and UV and γ-survival were assayed as described previously^10^. For the construction of N1, primers inCinA (5′-TGCTGTTTGGAGAAATCGTG-3′) and pastRecA (5′-GGGCAGCTCAAGACGTAAAA-3′) were used in conjunction with the Phusion Hot Start II High-Fidelity DNA Polymerase for PCR-amplification of the *recA* cassette which was subsequently used for the transformation of the pre-prepared CaCl2-competent wt cells^13^. Transformants were selected on the TGY plates supplemented with tetracycline, and confirmed with PCR and radiation-sensitivity. Bacteria were grown in TGY broth (0.5% tryptone, 0.1% glucose, 0.3% yeast extract) with aeration at 30 °C or on TGY plates with 1.5% agar.

### DNA extraction for sequencing

DNA was extracted by the phenol–chloroform protocol. Overnight cultures were pelleted and resuspended in 20 ml 95% ethanol and left for 10 min at room temperature. The cells were then pelleted again, resuspended in lysozyme solution (2 mg/ml) and incubated for 30 min at 37 °C. Proteinase K solution (0.8 mg/ml proteinase K, 2% SDS, 0.1 M EDTA) was added next, followed by vortexing and incubation for 3 h at 50 °C. Phenol–chloroform (1:1) was then added to the cell lysate, gently mixed and centrifuged at 12,000 rpm for 12 min to achieve the separation of phases. The water phase containing the DNA was then transferred into a new tube. Chloroform-isoamyl alcohol (24:1) and centrifugation was then used to remove the traces of phenol from the water phase. DNA was sodium-acetate precipitated and resuspended in milliQ water. The quality and quantity of DNA isolates were checked by spectrophotometric and agarose gel analysis.

### Sequencing, de novo assembly, and polishing of genome sequences

DNA was sent to a sequencing service (GATC PacBio service for samples wt, S1 and S2, Macrogen PacBio service for samples R1, R2 and R6). Details of DNA preparation and sequencing procedures performed by the sequencing services are listed in Table S1. Both raw data in h5 format, and subreads in fasta and fastq formats were obtained from the sequencing services. Statistics for obtained subreads are shown in Table S2. Subreads were used for de novo genome assemblies by the Canu assembler v.1.7^35^. The obtained contigs are listed in Table 1. Previously sequenced *D. radiodurans* wt genomes described by White et al. 1999^36^ (wt-1999) and Hua and Hua 2016^37^ (wt-2016) were used for comparisons throughout the paper (Table 1). Contigs were aligned to the wt-1999 with mummer, visualized with mummerplot^38^ and identified as *D. radiodurans* genome elements on the basis of homology (Table 1). All four genome elements were recovered for each sequenced *D. radiodurans* isolate (Table 1).

**Table 1:** GenBank accession numbers and sizes of contigs assembled by Canu for reference wild type (wt) and *recA* isolates, in comparison to the previously sequenced *D. radiodurans* wt genomes described by White et al. 1999 (wt-1999 sequence) and Hua and Hua 2016 (wt-2016 sequence). For the latter sequence, we report the length from the beginning of the reported sequence to the point where the beginning of the reported sequence starts again (i.e. we report the circularized genome element lengths). The identity of contigs as chromosomes (chr) I and II, and plasmids MP1 and CP1 was obtained through homology to the wt-1999 genome elements.

Strain/genome element	wt-1999 sequence	Length (bp)	Reference wt (this paper)	Length (bp)	S1 (*recA* isolate)	Length (bp)	S2 (*recA* isolate)	Length (bp)
Chr I	AE000513.1	2,648,638	CP038663	2,644,543	CP038975	2,647,698	CP038979	2,645,322
Chr II	AE001825.1	412,348	CP038664	412,189	CP038976	348,240	CP038980	360,746
MP1	AE001826.1	177,466	CP038665	177,363	CP038977	177,364	CP038981	177,363
CP1	AE001827.1	45,704	CP038666	45,503	CP038978	45,503	CP038982	46,549

Strain/genome element	R1 (*recA* isolate)	Length (bp)	R2 (*recA* isolate)	Length (bp)	R6 (*recA* isolate)	Length (bp)	wt-2016 sequence	Length (bp)
Chr I	CP038983	2,645,385	CP038987	2,646,569	CP038991	2,645,384	CP015081.1	2,646,741
Ch II	CP038984	334,190	CP038988	324,414	CP038992	376,662	CP015082.1	412,146
MP1	CP038985	177,304	CP038989	177,364	CP038993	177,364	CP015083.1	177,354
CP1	CP038986	43,155	CP038990	45,503	CP038994	51,683	CP015084.1	45,481

The Canu contigs corresponding to the *D. radiodurans* genome elements were not circularized by the assembler—the beginning of the sequence was repeated at the end of the contig (except for the plasmid MP1 from isolate R6). This also held true for some wt-2016 genome elements. Therefore, the genome elements were circularized by cropping the ending repeat, location of which was found with blastn^39,40^. To simplify subsequent genome comparisons, some of our genome elements, as well as the genome elements from wt-2016 were rewritten as their reverse complement to achieve the same directionality as the wt-1999 sequences. Additionally, their start positions were moved to correspond to the start positions of the wt-1999 genome elements. Therefore, the bp coordinates in the reference wt sequence reported in this paper roughly correspond to those in the wt-1999 sequence.

Two contigs in the isolate R1 and one contig in the isolate R6 were too short to correspond to the genome elements of the *D. radiodurans* genome sequence (3598, 3156 and 5977 bp, respectively). Blastn of the contig tig00000035 from R1 and contig tig00000027 from R6 against the nr/nt database matched these sequences to the parts of the “synthetic construct PacBio unrolled DNA internal control sequence” (GenBank accession MG551957.1). As for the contig tig00000003 from isolate R1, its two halves matched two consecutive sequences of opposite directionality on the chromosome I assembly of the R1 isolate. The directionality found within our assembly of chromosome I is supported by the corresponding sequences within chromosomes I from wt-1999 and wt-2016 (checked with Blast). Additionally, the contig tig00000003 from isolate R1 is only weakly supported by data—it is based on 5 PacBio reads. Therefore, we have excluded these three extra contigs from the subsequent analyses.

The assembled genomes were polished with Arrow. Briefly, bam files of subreads were obtained from the three .h5 files with bax2bam v.0.0.8 program. Pbmm2 v.0.8.1, a wrapper for Minimap2, was used to align the corresponding subreads to each assembly. The resulting bam files were sorted with Samtools v.1.9^41^ VariantCaller v.2.3.2 with arrow algorithm was called on the sorted bam files. The programs were installed via Miniconda 3 (obtained from https://conda.io/miniconda.html) configured to use defaults, conda-forge and Bioconda^42^ channels. The obtained genome assemblies have been deposited in GenBank and are available under accession numbers specified in Table 1.

### Data analysis

DNAdiff v.1.3 program from the Mummer package^38,43^ detects both structural rearrangements and single nucleotide polymorphisms (SNPs) and was used for genome comparisons. SNPs reported by DNAdiff may include small indels. *D. radiodurans* R1 sequences from the literature (wt-1999 and wt-2016, accession numbers in Table 1) are from strains ATCC BAA-816 and 13939 and have, presumably, diverged during propagation in different laboratories. Therefore, as the laboratory strains can differ, we sequenced our own wild type (here from referred to as reference wt) which was used for the construction of the *recA* mutants. Our reference wt corresponds to the ATCC 13939 strain by origin and is indeed more similar to the wt-2016 strain (we detected 160 SNPs with DNAdiff) than to the wt-1999 strain (559 SNPs). wt-1999 strain was, nevertheless, used throughout the paper to examine the genes affected by genome changes in our strains because the genes of this strain have been better characterized. To identify the genome changes (structural changes and SNPs) and their positions in *recA* mutants, the genome assemblies of *recA* isolates were compared to the reference wt assembly with DNAdiff. The *recA* deletion and its replacement with the antibiotic cassette was detected as a structural change in all the *recA* isolates and excluded from the downstream analyses. Additionally, large deletion and duplication were identified in the plasmid CP1 of the *recA* isolate R6, and a small deletion (~ 2 kb) in the plasmid CP1 of the isolate R1. However, these deletions were not confirmed with the lack of read coverage; a possible misassembly was implicated. Indeed, an alternative assembly obtained with the Miniasm assembler^44^ did not confirm these rearrangements; therefore, they were excluded from this report.

Known repetitive sequences were searched for at the rearrangement breakpoint areas. *D. radiodurans* insertion sequences were downloaded from ISfinder^45^. Additionally, shorter *D. radiodurans* repetitive sequences (SRE and SNRs) were obtained from Makarova et al^8^. Oligonucleotide repeats finder (developed by Bazin, Kosarev, Babenko) was used for exploration of rearrangement breakpoint areas, as well as the local alignment (blastn) of rearrangement carrying part of the *recA* genome and the corresponding non-rearranged reference. Breakpoint junction reconstruction (breakpoint repeat ± 60 bp) was performed for all the deletions in *recA* isolates and aligned to the region affected by deletion to confirm detected repeats at breakpoints. COG categorization of genes^46^ was obtained from the RefSeq ptt file. Repseek program^47^ was used for the abundance analysis of short repetitive sequences (briefly, we detected all the pairs of 100% identical sequences of specified length within the wt-1999 and, by taking into account repetitive sequence positions, we calculated the %coverage of the genome). INCA^48^ was used for the calculation of codon usage bias and %GC (including “GC3s”, i.e. %GC at 3^rd^ sites of fourfold degenerate amino acids) of the genome regions.

## Results and discussion

### Gross genome rearrangements in *D. radiodurans recA* isolates are deletions

Five *D. radiodurans recA* isolates carrying different genome rearrangements were selected based on the genome restriction patterns in PFGE gels (Fig. 1, also see “Methods”). These isolates were chosen among larger number of *recA* cultures to represent five continuously re-occurring rearrangement patterns within the *recA* population (^10^; our unpublished data). The selected *recA* isolates were sequenced and their genomes assembled in order to characterize the rearrangements (Table 1).

**Figure 2:**
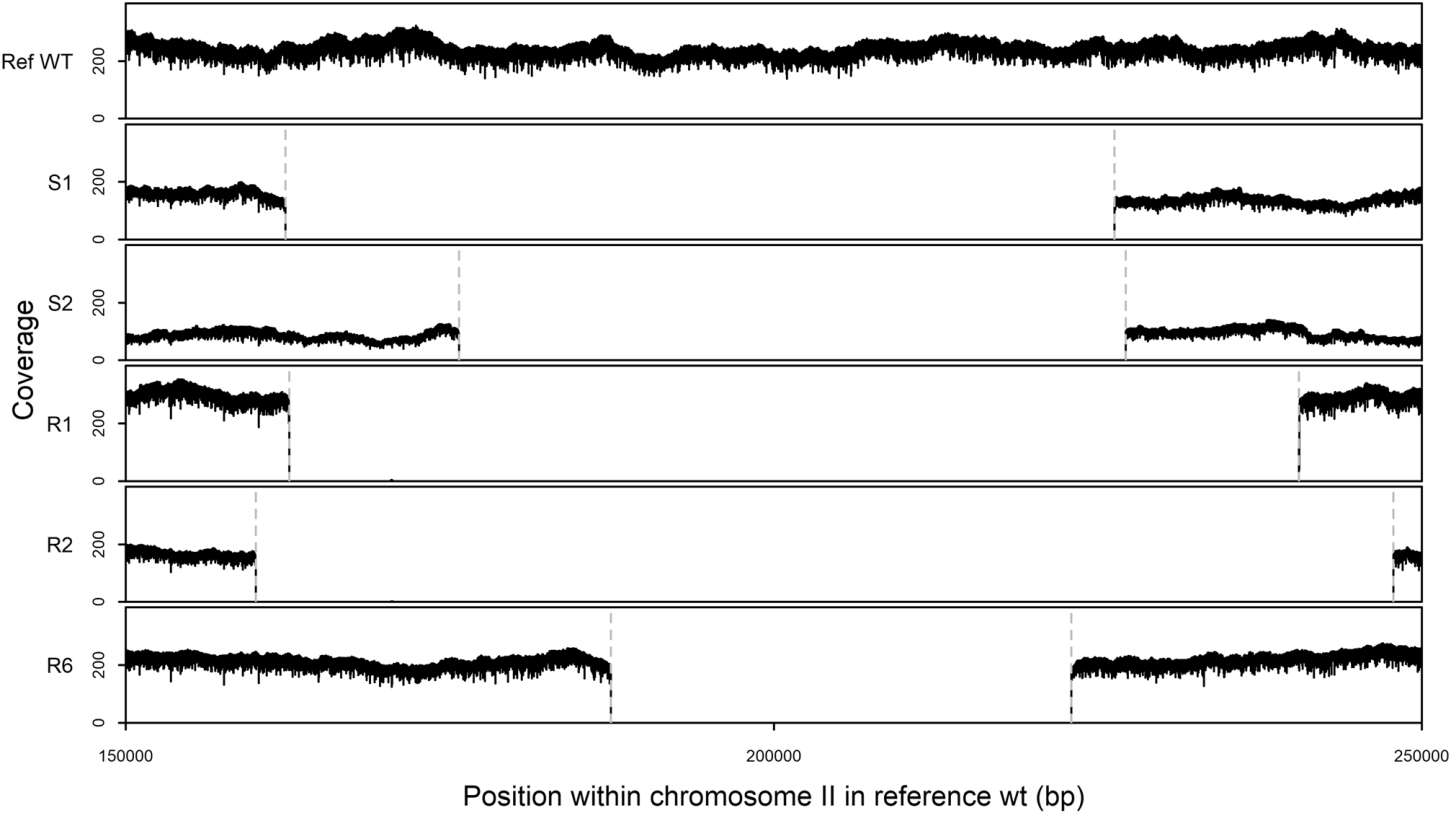
Sequencing read coverage of the chromosome II region affected by deletions in different *D. radiodurans recA* isolates. Vertical dashed lines correspond to the deletion borders identified in *recA* assemblies when compared to reference wt (bp coordinates in Table 2).

The genome rearrangements visible in the PFGE gels were identified as large deletions in chromosome II; each *recA* isolate carries a deletion spanning 8.6–21.3% of chromosome II (Table 1), a notable loss of coding DNA sequence. Large deletions we detected in the *recA* isolates occurred in a similar region of the *D. radiodurans* genome (Fig. 2)—the region of chromosome II between coordinates 160,030 and 247,789 bp (coordinates are given with respect to the reference wt).

Deletions and their exact coordinates were confirmed by aligning the raw sequencing (sub)reads to the reference wt genome; lack of reads in a genomic region signifies a genome deletion (Fig. 2). Lack of reads mapping to deleted chromosome II regions was evident in the PacBio data from all *recA* isolates. After our filtering for reliability (such as tests described in Fig. 2 and the “Methods” section under “Data analysis”), we found no gross genome rearrangements (deletions, duplications, inversions, insertions, translocations) other than the deletions described above. This might partly be due to the NotI-PFGE preselection of samples which might have limited the size and/or type of rearrangements we detected; this system of preselection is good for the detection of long (at least several tens of kilobases) deletions, duplications and insertions/translocations, as well as some rearrangements that span a NotI-restriction site. However, even a very large (e.g. hundreds of kilobases) inversion that happened within a 300 kb NotI-restriction fragment, as well as shorter genome rearrangements, could easily escape notice in this system.

IS sequence transpositions, presumably mediated by transposons, are relatively common in *D. radiodurans*^49^ and were detected in this study (Table S3) but excluded from downstream analyses, as these events are not relevant for the study of RecA-independent DNA repair.

### *D. radiodurans* utilizes short exact DNA repeats for DSB repair in the absence of RecA

We characterized the borders of genome rearrangements, i.e. the rearrangement breakpoints, in *recA* isolates to elucidate the underlying RecA-independent DNA repair mechanism. Short repeats of lengths 4–11 bp were detected at deletion breakpoints (Table 2), when comparing *recA* isolates to the reference wt.

**Table 2:** Coordinates (in bp) of *recA* deletions in *D. radiodurans* reference wt chromosome II, located with DNAdiff program, and direct repeats found at breakpoints. Start and end of deletion are reported as starting at the beginning of the repeat found at breakpoint. Local DNA sequences surrounding the repeats at breakpoint sites are presented in Table S5.

RecA isolate	Start of deletion (reference wt)	End of deletion (reference wt)	Length of deletion (bp)	Start of deletion (wt White et al. 1999)	End of deletion (wt White et al. 1999)	Repeat at breakpoints
S1	162,314	226,262	63,948	162,302	226,250	CGAGTTCGCGC
S2	175,704	227,142	51,423	175,692	227,130	CAGCC
R1	162,602	240,541	77,912	162,590	240,528	CGCCC
R2	160,014	247,789	87,759	160,002	247,776	CGATGG
R6	187,421	222,947	35,522	187,407	222,934	GGCA

We confirmed it was short exact repeats at deletion breakpoints as opposed to slightly longer inexact repeats by comparing the expected breakpoint sequences with the actual breakpoint sequences of *recA* isolates. A deletion between two repeats is expected to delete all the DNA between the repeats, as well as one of the (exact or inexact) repeat copies. We re-enacted the deletions between the short exact repeats in silico for all the five *recA* isolates; from the reference wt sequence we excised the DNA between each two exact repeats (repeat coordinates for each of the five isolates shown in Table 2), as well as one of the repeats. Each of the five resulting sequences carrying the in silico deletion was aligned to the one obtained by sequencing and assembling the corresponding *recA* isolate. We ascertained through this alignment that these in silico deletions at exact repeats exactly matched the breakpoint zones of the *recA* isolates, in all the five deletion re-enactments. We conclude from this comparison of expected and actual deletion breakpoints that exact repeats were used by the DNA repair mechanism that caused deletions.

The choice of repeats as substrates for DNA repair did not depend on the repeat nucleotide sequence; very different repeats were used for DNA repair in different rearrangement events (Table 2). Previously, genome rearrangements at much longer repeats (of lengths 438–3900 bp) have been detected in *D. radiodurans*^10,12,17^. Given these previous data, as well as the abundance of insertion sequences and other longer repeats in the *D. radiodurans* genome^8,9^, the discovery of short repeats at deletion break points in this work was somewhat surprising. Nevertheless, this discovery broadens the pool of known substrates for RecA-independent DNA repair in *D. radiodurans*.

The *recA*-independent mechanism of DSB repair detected in this work differs from SSA examples demonstrated previously in *D. radiodurans*^12,17^ in that it uses short exact repeats (4–11 bp) for DNA end attachment at DSBs. Given that SSA mechanism typically uses repetitive sequences more than 25 bp in length^50,51^, deletions between direct repeats as short as those detected here do not point unambiguously toward SSA and require consideration of other DNA repair mechanisms. Classical NHEJ, as an alternative to SSA, enables the direct ligation of two DSB ends sharing little or no homology^18,19^. When NHEJ involves homologies, they are usually up to 4 bp long, which is the length range that marginally overlaps with length range of repeats identified in this work. Despite this overlap, there are several arguments to dismiss NHEJ as the mechanism responsible for genomic rearrangements in our experimental system. First, classical NHEJ that operates in eukaryotes strongly requires Ku70 and Ku80 proteins to recognize DSB and to recruit other proteins needed to promote the joining of DNA ends^18,19^. The homologs of Ku proteins are also present in NHEJ-proficient bacterial species^20^ but not in *D. radiodurans*^9^. Second, NHEJ is basically homology (and sequence)-independent mechanism, and that fact is in contradiction with finding that all genome rearrangements detected in our study involved pre-existing repetitive sequences. Third, NHEJ is an error-prone mechanism that often causes loss or addition of bases when two DNA ends are joined^18^. However, in all *recA* mutants analysed in our work short repeats present at deletion breakpoints showed no sequence changes in comparison to their parental duplicates present prior to recombination. Such precision of DNA end joining is therefore more compatible with a SSA-like mechanism. Finally, in contrast to SSA, NHEJ does not involve significant DNA end resection, and therefore, it is not intrinsically prone to large chromosome deletions. Thus, it is not likely that DNA repair via NHEJ would engage two repetitive sequences separated by tens of kbp. This is particularly true for spontaneously rearranged *recA* strains (S1 and S2 in Table 1) where low co-occurrence of DSBs is expected.

The third mechanism that should be considered in the framework of our results is the alternative end-joining (A-EJ) that is also referred to as microhomology-mediated end-joining (MMEJ)^51–53^. This mechanism has been discovered and extensively studied in eukaryotes, but is also found in *E. coli*^54^. A-EJ (or MMEJ) is initially thought to act only as a back-up repair pathway, however later studies show that it is used even in the presence of functional HR and NHEJ, and it seems to become especially relevant in HR-defective backgrounds (see^53^ for a review). The A-EJ requires microhomology ranging from 2 to 20 bp in length^51^, and in that respect, it is a perfect candidate for the mechanism underlying the rearrangements observed in our study. Also, A-EJ mechanism does not depend on Ku proteins and other enzymes that are specifically required for NHEJ^51^. In fact, Ku proteins act as suppressors of A-EJ in eukaryotes^50^ so that the absence Ku homologs in *D. radiodurans* could be considered as condition that could potentially promote A-EJ in this bacterium. A-EJ, like SSA, requires end resection during DSB repair and annealing of the homologous parts of ssDNA overhangs created by the resection^51^. In principle, the resection step in both A-EJ and SSA could be carried out by the same enzymes (helicases and nucleases) that have been previously implicated in initiation of RecA-dependent homologous recombination in *D. radiodurans*^55,56^. The final DNA ligation step could be catalysed by NAD^+^-dependent DNA ligase (DRLigA)^57^ whose homolog was found to be required for A-EJ in *E. coli*^54^. Further experiments are necessary to identify which enzymes are involved in the *recA*-independent mechanism of DSB repair described in this work and to ascertain the degree of overlap in enzyme usage between different DSB repair mechanisms present in *D. radiodurans*.

Chromosome deletions revealed in this work in several aspects resemble A-EJ-mediated deletions observed in *E. coli*; (i) in both cases the deletions occurred in RecA-independent manner involving short direct repeats, (ii) the underlying mechanism works over large DNA linear distances, and (iii) the sequences bordering deletions remain unchanged. These findings strongly suggest that *D. radiodurans* and *E. coli* share the same RecA-independent DSB repair mechanism.

### Why do gross deletions recur in the same region of chromosome II?

Notably, all the large deletions we detected in the five *recA* isolates occur in the chromosome II of *D. radiodurans* (Fig. 2). Moreover, they occur in a similar region of the chromosome II.

Several explanations might account for the recurrence of deletions within a similar region of chromosome II. Firstly, the deletion recurrence could be coincidental. However, frequent recurrence of similar NotI-restriction patterns among the *recA* isolates (^10^, our unpublished results) makes this explanation unlikely. Secondly, selection might influence the frequency of particular deletions. For example, increased frequency of a deletion might be the consequence of positive selection for genome changes that confer an “improved” phenotype (e.g. faster growth or better oxidative stress survival). On the other hand, negative selection against some or most of deletions might reduce the variability of deletions present in a population. Thirdly, the recurring deletions could be mechanistically driven, by e.g. repeat density. Through sequence analysis of the five rearranged *D. radiodurans recA* isolates, we tested probable adaptive and mechanistic hypotheses that might explain the recurrent deletions of the same chromosome II region.

#### Evidence for positive selection affecting the recurrence of deletions

A 35 kb region deleted in all the sequenced isolates was used to assess the positive selection hypothesis which postulates that detected deletions might have a beneficial effect on phenotype. Due to the overlap of the deleted chromosomal areas (Fig. 2), the same region of ~ 35 kb between coordinates 187,425 and 222,947 bp was deleted in all the five sequenced isolates. This 35 kb region provided a framework for testing the putative adaptive significance of deletions: if a deletion is selected because of its positive effect on phenotype, the deletion is expected to be present in all the isolates (true for the 35 kb deletion).

The five rearranged *recA* isolates sequenced and presented here have higher than baseline resistance to γ-radiation (Figure S1) as they have been chosen for this phenotype from the pool of isolates with similar PFGE rearrangement patterns. However, there are some similarly rearranged *recA* isolates that did not show higher γ-resistance^10^ suggesting that these particular deletions are probably not the cause of the improved radiation resistance of *recA* isolates. The improved radiation resistance may have been achieved through different modifications in different isolates or may have been affected by mutations other than deletions that are present in all the isolates. For example, there are 6 SNPs identified by comparison to the reference wt that are common to all the analysed *recA* isolates (Table S4), but their effect on phenotype is not known.

The 35 kb region does not seem to carry genes deletion of which might improve the resistance to γ-irradiation or growth of a *D. radiodurans* carrying *recA* mutation. Such deletion would be expected to confer an improved strategy for avoidance of chromosomal fragmentation^58^. Mostly, the functional groups of genes in the deleted region (Table 3, see Table S6 for the functions deleted in at least one of the *recA* isolates) belong to the “accessory”, non-essential, functional groups expected on secondary replicons^59^. They include Clusters of Orthologous Groups (COGs) such as T (Signal transduction mechanisms), Q (Secondary metabolites biosynthesis, transport and catabolism), I (Lipid transport and metabolism) and G (Carbohydrate transport and metabolism). While the “accessory” functions in the 35 kb region seem to be deletion-permissive, especially in the context of rich medium and optimal growth conditions, their deletion is not expected to affect chromosomal fragmentation.

**Table 3:** Annotations of genes within the region of chromosome II deleted in all the *D. radiodurans recA* isolates. Annotations have been obtained from the wt-1999 sequence on the basis of sequence homology.

Gene coordinates (bp) in chr II of wt-1999	Gene id	Gene product	COG functional class	COG functional class annotation
186,398..187486	DR_A0181	GGDEF family protein	T	Signal transduction mechanisms
187,602..188408	DR_A0182	Hypothetical protein	L	Replication, recombination and repair
188,559..189953	DR_A0183	Hypothetical protein	R	General function prediction only
190,143..191132	DR_A0184	Pyridoxamine kinase	H	Coenzyme transport and metabolism
191,098..192645	DR_A0185	Exopolyphosphatase	FP	Nucleotide transport and metabolism; inorganic ion transport and metabolism
192,795..193784	DR_A0186	Cytochrome P450, putative	Q	Secondary metabolites biosynthesis, transport and catabolism
194,287..197055	DR_A0188	Excinuclease ABC subunit A	L	Replication, recombination and repair
197,036..198550	DR_A0189	Ribosomal protein S12 methylthiotransferase	J	Translation, ribosomal structure and biogenesis
198,551..200122	DR_A0190	Hypothetical protein		–
200,157..201089	DR_A0191	Hypothetical protein	G	Carbohydrate transport and metabolism
201,063..201476	DR_A0192	Hypothetical protein		–
201,536..202249	DR_A0193	Phosphoglycerate mutase-like protein	G	Carbohydrate transport and metabolism
202,242..203402	DR_A0194	Hypothetical protein	R	General function prediction only
203,399..204235	DR_A0195	Short chain dehydrogenase/reductase family oxidoreductase	IQR	Lipid transport and metabolism; secondary metabolites biosynthesis, transport and catabolism; general function prediction only
204,324..205571	DR_A0196	Acyl-CoA dehydrogenase	I	Lipid transport and metabolism
205,568..205975	DR_A0197	Hypothetical protein		–
205,972..206466	DR_A0198	Hypothetical protein	Q	Secondary metabolites biosynthesis, transport and catabolism
206,499..206960	DR_A0199	Nodulation protein N-like protein	I	Lipid transport and metabolism
206,957..207733	DR_A0200	Gluconate 5-dehydrogenase	IQR	Lipid transport and metabolism; secondary metabolites biosynthesis, transport and catabolism; general function prediction only
207,944..208807	DR_A0201	NAD synthetase	H	Coenzyme transport and metabolism
208,882..210270	DR_A0202	Cu/Zn family superoxide dismutase	P	Inorganic ion transport and metabolism
210,267..211598	DR_A0203	Oxidoreductase	G	Carbohydrate transport and metabolism
211,623..212054	DR_A0204	Response regulator	T	Signal transduction mechanisms
212,051..213649	DR_A0205	Sensor histidine kinase	T	Signal transduction mechanisms
213,762..215597	DR_A0206	Oligoendopeptidase F	E	Amino acid transport and metabolism
215,676..217316	DR_A0207	Hypothetical protein	S	Function unknown
217,422..218531	DR_A0208	Peptide ABC transporter permease	EP	Amino acid transport and metabolism; inorganic ion transport and metabolism
218,528..219514	DR_A0209	Peptide ABC transporter permease	EP	Amino acid transport and metabolism; inorganic ion transport and metabolism
220,213..221475	DR_A0210	Peptide ABC transporter, periplasmic peptide-binding protein	E	Amino acid transport and metabolism
221,435..222274	DR_A0211	GntR family transcriptional regulator	K	Transcription
221,476..223224	DR_A0212	Hypothetical protein	O	Posttranslational modification, protein turnover, chaperones

The putative benefit of the 35 kb deletion might be indirect as the loss of function mutations can sometimes have gain of fitness effects through metabolic and regulatory rewiring^60^. For example, inactivation of small metabolite transporters by the 35 kb deletion (Table 3) might have reduced the loss of metabolites, some of them potentially included in the scavenging of the reactive oxidative species in *D. radiodurans*. However, such possibility should be tested by additional experiments.

In all, there is no strong evidence for the beneficial effect of the 35 kb deletion.

#### Evidence for negative selection affecting the recurrence of deletions

The negative selection hypothesis postulates that different genome rearrangements can happen, but many of them are too deleterious to survive within the cell population. It is probable that the affected region of chromosome II is dispensable, especially in the conditions of rich medium and optimal growth conditions.

Surprisingly, functions of some genes within the 35 kb region were identified as potentially indispensable: DR_A0188 (*uvrA2*, expected to be involved in DNA repair), DR_A0202 (*sodC*, expected to be involved in the scavenging of oxidative radicals), and DR_A0189 (*rimO*, the ribosomal protein S12 methylthiotransferase) (Table 3). Due to the possibly important functions of these genes, the deletion of these genes was expected to have an adverse effect on growth and/or γ-survival. However, previous gene inactivation studies^61–63^ and gene distribution studies^64^ suggest that these genes don’t carry great importance for growth and/or radiation resistance. Altogether, the analysis of gene functions within the 35 kb deletion shows that gross deletions in chromosome II occur in a deletion-permissive region as there is no strong evidence for a putative deleterious effect of deletions.

There is no clear selection against deletions in other parts of chromosome II; large parts of the *D. radiodurans* genome are permissive to gross deletions, which, contrariwise, cluster within the same region of chromosome II. We have tested whether the region of chromosome II “targeted” by deletions is more deletion-permissive i.e. dispensable than other regions of chromosome II. If not, a mechanistic explanation for the deletion “targeting” may be in order. Genomic signatures, such as codon usage, can denote adaptive advantages. Weaker codon usage bias within the affected 35 kb region would point towards higher dispensability of its genes and vice versa. We detected no weaker codon usage bias in the genes of the deleted region when compared to the genes of the whole chromosome II (median synonymous codon usage order (SCUO) was 0.3831 for the genes belonging to the deleted region and 0.3543 for all the chromosome II genes—lower SCUO signifies lower codon usage bias^65^). This points towards similar dispensability of genes in different regions of chromosome II. The differences in the background GC composition might affect the codon bias measurements. However, GC composition of the affected region was similar to the GC composition of the whole chromosome II (67.4% GC and 66.7% GC, respectively). Another measure of the background composition, the GC composition at silent sites of codons (i.e. at 3rd codon positions) is also similar when comparing the affected region and the whole chromosome II (91% and 88%, respectively).

In all, other regions of chromosome II seem to be equally susceptible to deletions as the affected region, ruling against negative selection as a sole explanation for deletion recurrence.

#### Evidence for mechanistic drive behind the recurrence of deletions

##### Occurrence of repeated sequences does not explain the recurrence of deletions in chromosome II

Of all the *D. radiodurans* genome elements, chromosome II contains the lowest number of IS copies per 10,000 bp^8^. Moreover, our results show that the repeats used as substrates for RecA-independent DNA repair might be very short (4–11 bp, Table 2). The abundance of such short repeats in the whole *D. radiodurans* genome is very high—we calculated the genome coverage for 11 bp repeat pairs to be 100% (see “Methods”). Therefore, the deletions don’t seem to have been caused specifically by repeat occurrence.

##### There is no support for active targeting of deletions

The deletions are unlikely to have been the consequence of an active targeting mechanism; they greatly differ in size, and their breakpoints are (mostly) very distant from each other. Further eroding support for an active targeting mechanism, breakpoints and their surrounding sequences are very different from each other (Table S5).

##### Frequency of DSBs (and dispensability) favours deletions in chromosome II

A set rate of DSBs per Mbp, whether low (e.g. during spontaneous growth that resulted in isolates S1 and S2) or high (e.g. after acute γ-radiation that resulted in isolates R1, R2 and R6) is more likely to affect chromosomes than plasmids, due to their size. A primary replicon, such as *D. radiodurans* chromosome I, carries most of the essential genes. A secondary replicon, such as *D. radiodurans* chromosome II typically carries some essential genes, but mostly accessory genes important when changing environments^66^. Plasmids don’t carry essential genes, by definition^59^. Plasmids, while potentially permissive to deletions, as well as chromosome II, are less likely to suffer DSBs. Therefore, chromosome II is the most frequent “target” of DSBs while also sporting dispensable DNA regions.

##### The region of deletion in chromosome II coincides with the region of replication termination

The DoriC database^67,68^ predicts the position of the origin of replication close to coordinate 1 bp in the chromosome II. Based on the length of the chromosome II and the presumption of replichore balance, terminus of replication is situated around coordinate 206,000 bp. Hence, the terminus-related sequences of chromosome II between coordinates 187,425 and 222,947 bp seem to have been deleted in all the *recA* isolates. Research on *E. coli* has revealed that termination of replication is a rather complex process that should be tightly regulated in order to avoid potentially lethal DNA transactions^69^. Replication fork collision in the terminus region may result in single-stranded and double-stranded DNA ends that instigate recombination^69^. In *recA* mutants, such ends could be directed toward non-homologous (illegitimate) recombination pathways. Additional challenge to the stability of the terminus region may come from spontaneous DSBs associated with collapse of the replication forks. Although such DSBs may arise anywhere in the genome, the convergence of replication forks in terminus region increases the probability for DNA breaks to co-occur in relative proximity. Therefore, even in the absence of γ-radiation assault, the combined higher genome instability resulting from the *recA* genotype and convergence of replication forks might facilitate deletions in the replication-termination region of chromosome II.

## Conclusion

*D. radiodurans recA* isolates carrying gross genome rearrangements were sequenced and their genomes fully assembled de novo with the goal of identifying genome rearrangements and characterizing the *D. radiodurans *in situ RecA-independent DSB repair. The detected rearrangements consisted of large deletions in chromosome II in all the sequenced *recA* isolates. Characteristics of the detected DSB repair differed significantly from the SSA repair previously demonstrated in *D. radiodurans*; the detected DSB repair utilized short repeats as opposed to otherwise abundantly present long repeats and worked over larger linear DNA distances from those previously tested. We detected no sequence changes in regions bordering large deletions, i.e. no proof of a NHEJ mechanism, in concordance with literature. Our results suggest that large genome deletions in *D. radiodurans recA* mutants occur via alternative end-joining (A-EJ) that mechanistically resembles SSA. All the deletions were situated in a similar region of chromosome II, likely due to a combination of several factors: (i) negative selection for rearrangements in other genome regions, (ii) higher occurrence or co-occurrence of DSBs at the terminus region of chromosome II resulting from both the *recA* genotype and convergence of replication forks, and (iii) negative filtering of isolates possibly carrying smaller-scale genome rearrangements (due to limitations of PFGE as a method for rearrangement detection). Except for the genome rearrangements described above, we found no evidence of other rearrangements in the five sequenced strains. However, our PFGE system for rearrangement detection might have missed clones carrying small scale and/or lethal rearrangements caused by mechanisms other than A-EJ.

The conclusions of our study are limited by the type of experiments we have done. We detect a new DSB repair mechanism in *D. radiodurans*, but its exact identification relies on matching a limited set of the detected characteristics with characteristics typical for potential mechanisms reported in the literature. Even though reported characteristics of A-EJ best match the observations, additional work is needed to delineate possible functional overlaps or cross-talk with other DNA repair mechanisms, and identify enzymatic functions involved. Our experiments could only detect A-EJ through genome rearrangements; unexpectedly, all the detected rearrangements occurred in the similar region of chromosome II, on which non-essential functions tend to be coded. Further experimentation is needed to confirm whether other genomic changes could be associated with the novel mechanism, and whether other genome regions are susceptible to these changes.

Our previous and present results are the first to demonstrate large DNA rearrangements involving only genome sequences naturally present in *D. radiodurans* cells (Repar et al. 2010^10^; this paper). In addition, all the detected rearrangements were observed in living cells thus implying that the underlying A-EJ mechanism contributes to cell survival through DSB repair. Although this contribution might appear negligible compared to that of the RecA-dependent repair mechanisms, the A-EJ pathway may provide a significant add to the survival kit of *D. radiodurans*, especially when combined with an effective antioxidation protein-protection that is also present in this bacterium^23–25^. Indeed, *D. radiodurans* lacking *recA* is similarly radiation resistant as wild-type *E. coli*^70^ suggesting that under the conditions of antioxidation protein-protection, RecA-independent DNA repair mechanisms, such as SSA and A-EJ, can significantly contribute to radiation survival.

## Supplementary Information

Supplementary Information.

## Data Availability

Assembled genomes have been submitted to NCBI Genbank (see Table 1 for accession numbers).
