# Supplementary material for: Characterization of gross genome rearrangements in Deinococcus radiodurans recA mutants
Source: Sci Rep. 2021 May 25;11:10939. doi: 10.1038/s41598-021-89173-9 (PMC8149714; doi:10.1038/s41598-021-89173-9)
Supplement: Supplementary file 1 — Supplementary Information. [file 41598_2021_89173_MOESM1_ESM.pdf]

**Title**

# Characterization of gross genome rearrangements in *Deinococcus radiodurans recA* mutants

**Authors**: Jelena Repar[1*], Davor Zahradka[1], Ivan Sović[2], Ksenija Zahradka[1*]

[1]Laboratory for Molecular Microbiology, Division of Molecular Biology, Ruđer Bošković Institute, Bijenička cesta 54, 10000 Zagreb, Croatia

[2]Digital BioLogic d.o.o, Ivanić-Grad, Croatia

*Corresponding authors: Jelena.Repar@irb.hr, Ksenija.Zahradka@irb.hr, Ruđer Bošković Institute, Bijenička cesta 54, 10000 Zagreb, Croatia, Tel: +38514560971, Fax: +38514561177

**Supplementary data**

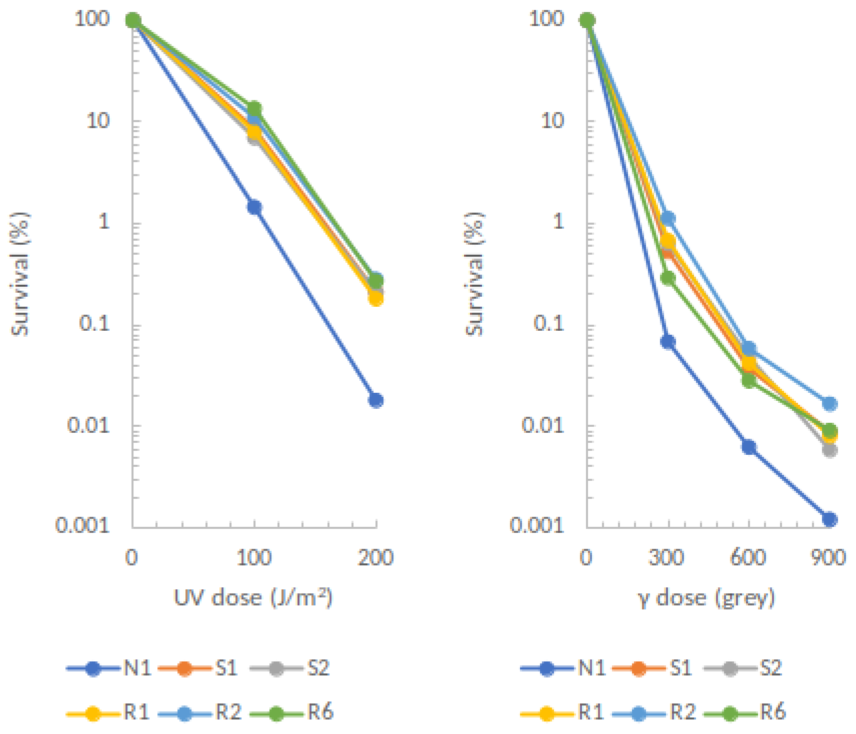


**Figure S1.** Representative survival curves after UV and γ-radiation for different *recA* isolates. N1 is the control *recA* strain, S1 and S2 are *recA* isolates carrying spontaneous DNA rearrangements accumulated during normal lab propagation, and R1, R2 and R6 are *recA* isolates carrying DNA rearrangements accumulated after γ-radiation (see Materials and Methods).

**Table S1.** Preparation of samples for sequencing by the sequencing services

| Sequencing service | Reagent (Binding Kit) | Sequencing protocol (Template Prep) | Chemistry | Insert Size (bp) |
|---|---|---|---|---|
| GATC | DNA/Polymerase Binding Kit P6 | DNA Template Prep Kit 2.0 | P6-C4 | 20000 |
| Macrogen | DNA/Polymerase Binding Kit P6 v2 | DNA Template Prep Kit 3.0 | P6-C4 | 20000 |

**Table S2.** Subread statistics for different strains sequenced by PacBio. Genome used for the calculation of coverage is the White et al 1999 *Deinococcus radiodurans* sequence.

| | wt | S1 | S2 | R1 | R2 | R6 |
|---|---|---|---|---|---|---|
| Mean Subread Length | 6409 | 6115 | 5942 | 10765 | 7532 | 8575 |
| Total Number of Bases | 1224556612 | 723333716 | 376472199 | 1347048152 | 802228016 | 767490527 |
| Subread N50 | 8322 | 8014 | 7630 | 16822 | 11347 | 12860 |
| Number of reads | 191042 | 118283 | 63350 | 125131 | 106499 | 89494 |
| Coverage of reference | 373 | 220 | 115 | 410 | 244 | 234 |

**Table S3.** Transposition events identified in *recA* isolates. All the listed coordinates are within chromosome I of the specified *recA* isolates. Transposed sequences were blastn-aligned to the known *D. radiodurans* IS sequences from ISFinder database for identification.

| *recA* isolate | Coordinate I (bp) | Coordinate II (bp) | Length of duplication | Similarity to known *D. radiodurans* ISes |
|---|---|---|---|---|
| S1 | 626699 | 628020 | 1322 | IS2621 |
| S2 | 8437 | 9486 | 1050 | ISDra4 |
| R2 | 1572426 | 1573603 | 1178 | - |

**Table S4.** Mapping of the SNPs common to all the *recA* isolates to the annotated genes in the wt *D. radiodurans* (White et al. 1999). There are 6 SNPs identified by comparison to the reference wt that are common to all the sequenced *recA* isolates, and that are also confirmed by comparison to the ref-2016 (same strain as reference). They are all located on chromosome I. Two of the common SNPs are in the intergenic regions (locations 394777, 1112537 in wt-1999) and two are in *recA* operon, within genes considered inactivated by *recA* deletion construction and are, therefore, unlikely to have phenotypic consequences, and the rest are described in the table.

| Genome element | Coordinate of the SNP (bp) | Gene coordinates (bp) | Gene id | Gene product | COG functional class | COG functional class annotation |
|---|---|---|---|---|---|---|
| AE000513 | 669378 | 668543..669382 | DR_0657 | serine esterase | R | General function prediction only |
| AE000513 | 1350441 | 1350424..1351092 | DR_1344 | hydrolase | R | General function prediction only |

**Table S5.** Local DNA sequences found at deletion breakpoint sites

| *recA* isolate | Site of deletion start in reference wt | Sequence +- 30 bp around the repeat situated at the deletion start site | Site of deletion end in reference wt | Sequence +- 30 bp around the repeat situated at the deletion end site | Sequence after deletion (as found in the corresponding *recA* isolate) |
|---|---|---|---|---|---|
| S1 | 162314 | CCTGCGCGCCGGA<br>ACAGCCGGCCACC<br>CGTC**CGAGTTCGC**<br>**GC**AGGCGCTCGCG<br>CTGCTGCTGCAGAT<br>GGCG | 226262 | GCTCGAACGGGTGA<br>GCGGACGACCCCTG<br>GG**CGAGTTCGCGC**T<br>GGACGCGGAGCTGA<br>CCTTTGTTCCTGACC | CCTGCGCGCCGGA<br>ACAGCCGGCCACC<br>CGTC**CGAGTTCGC**<br>**GC**TGGACGCGGAG<br>CTGACCTTTGTTCC<br>TGACC |
| S2 | 175704 | TTCCGGACGGTGG<br>GTCGAGCGGCCCA<br>CCCG**CAGCC**CCGG<br>CGCCATGAGCCGC<br>GCCCACTCCACCG | 227142 | GGCGCGGCCCCGGT<br>GCAGCCCGCCCACC<br>CC**CAGCC**GCACCGG<br>CTGCCCGGTGGACTC<br>GGTGAGCG | TTCCGGACGGTGG<br>GTCGAGCGGCCCA<br>CCCG**CAGCC**GCAC<br>CGGCTGCCCGGTG<br>GACTCGGTGAGCG |
| R1 | 162602 | GCGCCTGCTCGCCC<br>GCCCGGCCCCGAG<br>CGA**CGCCC**CGGCC<br>TGAGCTTTCGCGCC<br>CCCACGTCCTC | 240541 | GCCTCCCTTGCCCGC<br>CAGGCCCCTGAGCG<br>C**CGCCC**GTTTGCCG<br>CCCTGGCCGTCGCGC<br>ACGTAC | GCGCCTGCTCGCCC<br>GCCCGGCCCCGAG<br>CGA**CGCCC**GTTTG<br>CCGCCCTGGCCGTC<br>GCGCACGTAC |
| R2 | 160014 | GGCGCGCGCTGGG<br>CGAAACGCTCGCC<br>GTGG**CGATGG**TCA<br>TCGGCGACAGCCA<br>GGACGTGATTCGC<br>A | 247789 | CCGCCAACTCGCAGT<br>TCATCGGCGGGATG<br>A**CGATGG**GCATCAG<br>CGCCGCGCTGCACG<br>AGGAAAGTT | GGCGCGCGCTGGG<br>CGAAACGCTCGCC<br>GTGG**CGATGG**GCA<br>TCAGCGCCGCGCT<br>GCACGAGGAAAGT<br>T |
| R6 | 187421 | CGTGGGCGACTGC<br>TTTCGACGGGCGG<br>ACGT**GGCA**CTTTA<br>CGCGGCGAAGCGA<br>GGAGGGCGGAAT | 222947 | GGCGGCGTGGTCGC<br>CTTCGACACCGGCCC<br>C**GGCA**ACTGCCTGC<br>TCGACGAACTTGCCG<br>GGCAGG | CGTGGGCGACTGC<br>TTTCGACGGGCGG<br>ACGT**GGCA**ACTGC<br>CTGCTCGACGAACT<br>TGCCGGGCAGG |

**Table S6.** Annotations of genes within the region of chromosome II deleted in at least one *D. radiodurans recA* isolate. Annotations have been obtained from the wt-1999 sequence on the basis of sequence homology.

| Gene coordinates (bp) in chr II of wt-1999 | Gene id | Gene product | COG functional class | COG functional class annotation |
|---|---|---|---|---|
| 159174..160223 | DR_A0158 | phosphate ABC transporter permease | P | Inorganic ion transport and metabolism |
| 160229..161125 | DR_A0159 | phosphate ABC transporter permease | P | Inorganic ion transport and metabolism |
| 161174..161938 | DR_A0160 | phosphate ABC transporter ATP-binding protein | P | Inorganic ion transport and metabolism |
| 161935..162603 | DR_A0161 | phosphate transport system regulatory protein PhoU | P | Inorganic ion transport and metabolism |
| 162820..163410 | DR_A0162 | hypothetical protein | R | General function prediction only |
| 163407..163880 | DR_A0163 | hypothetical protein | R | General function prediction only |
| 163921..164349 | DR_A0164 | thioredoxin 1 | O | Posttranslational modification, protein turnover, chaperones |
| 164630..164881 | DR_A0165 | hypothetical protein | / | / |
| 164884..169764 | DR_A0166 | hypothetical protein | / | / |
| 169827..170411 | DR_A0167 | hypothetical protein | S | Function unknown |
| 170655..171455 | DR_A0168 | molybdenum ABC transporter permease | O | Posttranslational modification, protein turnover, chaperones |
| 171517..172272 | DR_A0169 | molybdenum ABC transporter periplasmic molybdate-binding protein | P | Inorganic ion transport and metabolism |

| | | | | |
|---|---|---|---|---|
| 172369..173013 | DR_A0170 | hypothetical protein | / | / |
| 173457..174212 | DR_A0171 | phosphomethylpyrimidine kinase | H | Coenzyme transport and metabolism |
| 174209..175075 | DR_A0172 | thiazole synthase | H | Coenzyme transport and metabolism |
| 175100..175375 | DR_A0173 | hypothetical protein | H | Coenzyme transport and metabolism |
| 175372..176214 | DR_A0174 | thiamin-phosphate pyrophosphorylase | H | Coenzyme transport and metabolism |
| 176242..178065 | DR_A0175 | thiamine biosynthesis protein ThiC | H | Coenzyme transport and metabolism |
| 178521..179963 | DR_A0176 | xanthine permease | F | Nucleotide transport and metabolism |
| 180141..181595 | DR_A0177 | xanthine dehydrogenase, N-terminal subunit | F | Nucleotide transport and metabolism |
| 181592..184015 | DR_A0178 | xanthine dehydrogenase, C-terminal subunit | F | Nucleotide transport and metabolism |
| 184027..184851 | DR_A0179 | hypothetical protein | O | Posttranslational modification, protein turnover, chaperones |
| 184848..186167 | DR_A0180 | guanine deaminase | FR | Nucleotide transport and metabolism; General function prediction only |
| 186398..187486 | DR_A0181 | GGDEF family protein | T | Signal transduction mechanisms |
| 187602..188408 | DR_A0182 | hypothetical protein | L | Replication, recombination and repair |
| 188559..189953 | DR_A0183 | hypothetical protein | R | General function prediction only |
| 190143..191132 | DR_A0184 | pyridoxamine kinase | H | Coenzyme transport and metabolism |
| 191098..192645 | DR_A0185 | exopolyphosphatase | FP | Nucleotide transport and metabolism; Inorganic ion transport and metabolism |
| 192795..193784 | DR_A0186 | cytochrome P450, putative | Q | Secondary metabolites biosynthesis, transport and catabolism |
| 194287..197055 | DR_A0188 | excinuclease ABC subunit A | L | Replication, recombination and repair |
| 197036..198550 | DR_A0189 | ribosomal protein S12 methylthiotransferase | J | Translation, ribosomal structure and biogenesis |
| 198551..200122 | DR_A0190 | hypothetical protein | / | / |
| 200157..201089 | DR_A0191 | hypothetical protein | G | Carbohydrate transport and metabolism |
| 201063..201476 | DR_A0192 | hypothetical protein | / | / |
| 201536..202249 | DR_A0193 | phosphoglycerate mutase-like protein | G | Carbohydrate transport and metabolism |
| 202242..203402 | DR_A0194 | hypothetical protein | R | General function prediction only |
| 203399..204235 | DR_A0195 | short chain dehydrogenase/reductase family oxidoreductase | IQR | Lipid transport and metabolism; Secondary metabolites biosynthesis, transport and catabolism;General function prediction only |
| 204324..205571 | DR_A0196 | acyl-CoA dehydrogenase | I | Lipid transport and metabolism |
| 205568..205975 | DR_A0197 | hypothetical protein | / | / |
| 205972..206466 | DR_A0198 | hypothetical protein | Q | Secondary metabolites biosynthesis, transport and catabolism |
| 206499..206960 | DR_A0199 | nodulation protein N-like protein | I | Lipid transport and metabolism |
| 206957..207733 | DR_A0200 | gluconate 5-dehydrogenase | IQR | Lipid transport and metabolism; Secondary metabolites biosynthesis, transport and catabolism;General function prediction only |
| 207944..208807 | DR_A0201 | NAD synthetase | H | Coenzyme transport and metabolism |
| 208882..210270 | DR_A0202 | Cu/Zn family superoxide dismutase | P | Inorganic ion transport and metabolism |
| 210267..211598 | DR_A0203 | oxidoreductase | G | Carbohydrate transport and metabolism |
| 211623..212054 | DR_A0204 | response regulator | T | Signal transduction mechanisms |
| 212051..213649 | DR_A0205 | sensor histidine kinase | T | Signal transduction mechanisms |
| 213762..215597 | DR_A0206 | oligoendopeptidase F | E | Amino acid transport and metabolism |
| 215676..217316 | DR_A0207 | hypothetical protein | S | Function unknown |
| 217422..218531 | DR_A0208 | peptide ABC transporter permease | EP | Amino acid transport and metabolism; Inorganic ion transport and metabolism |
| 218528..219514 | DR_A0209 | peptide ABC transporter permease | EP | Amino acid transport and metabolism; Inorganic ion transport and metabolism |
| 220213..221475 | DR_A0210 | peptide ABC transporter, periplasmic peptide-binding protein | E | Amino acid transport and metabolism |
| 221435..222274 | DR_A0211 | GntR family transcriptional regulator | K | Transcription |

| | | | | |
|---|---|---|---|---|
| 221476..223224 | DR_A0212 | hypothetical protein | O | Posttranslational modification, protein turnover, chaperones |
| 223528..224445 | DR_A0213 | N-acetylmuramic acid-6-phosphate etherase | R | General function prediction only |
| 224467..225069 | DR_A0214 | trp repressor binding protein WrbA | R | General function prediction only |
| 225335..225730 | DR_A0215 | hypothetical protein | / | / |
| 225739..226740 | DR_A0216 | esterase | V | Defense mechanisms |
| 226744..227928 | DR_A0217 | hypothetical protein | TQ | Signal transduction mechanisms; Secondary metabolites biosynthesis, transport and catabolism |
| 227890..228774 | DR_A0218 | hypothetical protein | V | Defense mechanisms |
| 229381..230187 | DR_A0219 | hypothetical protein | / | / |
| 230198..231772 | DR_A0220 | aldehyde dehydrogenase | C | Energy production and conversion |
| 231769..232287 | DR_A0221 | glucan synthase 1-like protein | G | Carbohydrate transport and metabolism |
| 232284..233150 | DR_A0222 | TDP-glucose-4,6-dehydratase-like protein | MG | Cell wall/membrane/envelope biogenesis; Carbohydrate transport and metabolism |
| 233147..234661 | DR_A0223 | 4-hydroxyphenylacetate-3-hydroxylase | Q | Secondary metabolites biosynthesis, transport and catabolism |
| 234780..235802 | DR_A0224 | homoprotocatechuate 2,3-dioxygenase | E | Amino acid transport and metabolism |
| 235813..236883 | DR_A0225 | sulfite oxidase | R | General function prediction only |
| 236880..237668 | DR_A0226 | 2-hydroxyhepta-2,4-diene-1,7-dioate isomerase/5-carboxymethyl-2-oxo-hex-3-ene-1,7-dioate decarboxylase | Q | Secondary metabolites biosynthesis, transport and catabolism |
| 237755..238141 | DR_A0227 | 5-carboxymethyl-2-hydroxymuconate isomerase | E | Amino acid transport and metabolism |
| 238138..238491 | DR_A0228 | hypothetical protein | R | General function prediction only |
| 238531..239295 | DR_A0229 | hypothetical protein | / | / |
| 239292..239891 | DR_A0230 | hypothetical protein | / | / |
| 239989..242217 | DR_A0231 | oxidoreductase | C | Energy production and conversion |
| 242214..243248 | DR_A0232 | oxidoreductase | C | Energy production and conversion |
| 243245..243808 | DR_A0233 | oxidoreductase iron-sulfur subunit | C | Energy production and conversion |
| 243966..244139 | DR_A0234 | hypothetical protein | / | / |
| 244292..244936 | DR_A0235 | oxidoreductase iron-sulfur subunit | C | Energy production and conversion |
| 244933..245949 | DR_A0236 | oxidoreductase | C | Energy production and conversion |
| 245946..248072 | DR_A0237 | oxidoreductase | C | Energy production and conversion |
